# Cancer risk in individuals with psychiatric disorders: population-based cohort study

**DOI:** 10.1192/bjo.2025.783

**Published:** 2025-06-20

**Authors:** Tak Kyu Oh, Hye Yoon Park, In-Ae Song

**Affiliations:** Department of Anesthesiology and Pain Medicine, College of Medicine, Seoul National University, Seoul, Republic of Korea; Department of Anesthesiology and Pain Medicine, Seoul National University Bundang Hospital, Seongnam, Republic of Korea; Department of Psychiatry, College of Medicine, Seoul National University Hospital, Seoul, Republic of Korea

**Keywords:** Cancer, psychiatric disorder, depression, cohort studies

## Abstract

**Background:**

The risk of cancers associated with psychiatric disorders is understudied.

**Aims:**

To investigate whether cancer risk varies with the presence of psychiatric disorders.

**Method:**

Patients diagnosed with psychiatric disorders in South Korea between 1 January and 31 December 2017 were included in the study and referred to as the psychiatric disorder group. The non-psychiatric-disorder group, selected using a stratified random sampling technique based on age and gender, comprised individuals who had never been diagnosed with a psychiatric disorder. The primary outcome was a new cancer diagnosis, assessed over a 5-year period (1 January 2018 to 31 December 2022).

**Results:**

Following 1:1 propensity score matching, the final analysis included data for 686 570 adults (343 285 in each group). The cancer incidence in the psychiatric disorder group from 2018 to 2022 was 15.4% (52 948/343 285), whereas in the non-psychiatric-disorder group, it was 12.8% (43 989/343 285). Cox regression analysis revealed that the psychiatric disorder group had a 23% higher occurrence of cancer compared with non-psychiatric-disorder controls (hazard ratio: 1.23, 95% CI: 1.21, 1.24; *P* < 0.001). Significant associations between cancer incidence and specific psychiatric disorders were observed in individuals with alcohol-related disorders (hazard ratio: 1.27, 95% CI: 1.23, 1.32; *P* < 0.001), anxiety disorders (hazard ratio: 1.15, 95% CI: 1.14, 1.17; *P* < 0.001) and major depressive disorder (hazard ratio: 1.16, 95% CI: 1.15, 1.18; *P* < 0.001).

**Conclusions:**

Individuals with psychiatric disorders were more likely to develop cancer than those without. We identified associations of alcohol-related disorders, anxiety disorders and major depressive disorder with cancer risk.

Cancer represents the primary cause of death globally.^1^ Between 2006 and 2016, 17.2 million individuals were diagnosed with cancer, resulting in 8.9 million fatalities and representing a 28% increase in the total number of cancer cases.^2^ The number of new cancer cases per population is expected to gradually increase; therefore, it is important to implement prevention efforts to lessen the worldwide disease burden.^3^ Psychiatric morbidities are also prevalent in the general population,^4^ and a substantial body of literature indicates higher mortality rates among individuals with various types of psychiatric condition.^5^ The relationships observed between psychiatric disorders and cancer occurrence are variable, with cancer rates among individuals with psychiatric conditions reported to be higher, comparable with or lower than those in the general population.^6,7^ However, the choice of analytical approach significantly influences the observed relationship between psychiatric disorders and cancer occurrence, and this relationship is complex and varies over time.^8^

In previous studies, researchers have examined the risk of cancer development in patients with specific psychiatric conditions compared with those without such conditions.^6,7,9,10^ Nonetheless, it is important to consider that multiple psychiatric disorders frequently occur concurrently,^11^ and this may influence a patient’s risk of developing cancer. Furthermore, analysis of cancer risk in patients without mental illness necessitates more precise control groups and extended follow-up periods, aspects that previous studies have frequently overlooked.^6,7,9,10^ Consequently, there is an ongoing need for methodologically robust research on this subject. In the present study, we investigated the associations between underlying psychiatric morbidities and cancer incidence using a nationwide database in South Korea.

## Method

### Experimental design and ethical considerations

This retrospective population-based cohort study adhered to the Strengthening the Reporting of Observational Studies in Epidemiology (STROBE) guidelines.^12^ The authors state that all procedures used in this study were in accordance with the ethical requirements of the appropriate national and institutional human experimentation committees, as well as the Helsinki Declaration of 1975, as revised in 2013. Our institutional review board approved all procedures involving human participants and patients (approval number: X-2303-819-902). The Big Data Center of the National Health Insurance Service (NHIS) (NHIS-2023-1-526) authorised access to the data, including sharing rights. As the data were anonymised and the study was retrospective, it was not necessary to obtain informed consent from patients.

### NHIS database

This study used data sourced from the NHIS, a public insurance programme in South Korea. The NHIS database, as required by law, includes detailed records of all disease diagnoses and associated prescription information for treatments and medications. To be eligible for government-funded health insurance programmes, individuals must enrol in the NHIS, and all diagnoses are reported and categorised on the basis of the ICD-10. Individuals residing in South Korea for more than 6 months must complete their registration with the NHIS. Furthermore, the NHIS database offers precise information about socioeconomic status and date of death.^13^

### Study population

Data were initially obtained from the NHIS concerning adult patients (≥18 years old) diagnosed with psychiatric disorders and receiving treatment during the period from 1 January to 31 December 2017. The ICD-10 codes for the psychiatric disorders examined in this study are listed in Supplementary Table 1 available online at https://doi.org/10.1192/bjo.2025.783. Upon obtaining data indicating that the population exceeded 5 million, we requested a stratified 10% sample categorised by age and gender. In the present study, adults identified as having psychiatric disorders in 2017 were assigned to the psychiatric disorder group. Following identification of all individuals eligible for inclusion in this group in South Korea in 2017, we requested data for individuals who had never been diagnosed with psychiatric disorders, to form our non-psychiatric-disorder group. This was accomplished using a 1:1 stratified random sampling technique, considering both age and gender. Owing to tracking of cancer diagnoses from 2018 to 2022, individuals who died in 2017 were excluded from the study. Furthermore, individuals with a cancer diagnosis before 2018 were excluded, as the focus of the study was cancer cases diagnosed from 2018 onward.

### Experimental endpoints

The primary study outcome was new cancer diagnoses, assessed over a 5-year period from 1 January 2018 to 31 December 2022. Cancer diagnoses were made using ICD-10 codes (C00–C96). Patients with cancer in South Korea are registered in the NHIS database, which includes the assignment of a specific code known as the C code (i.e. a cancer diagnosis), entitling such individuals to unique insurance benefits. The following cancers were uniquely categorised: gastric cancer (C16), oesophageal cancer (C15), colorectal cancers (C18–C20), gallbladder and biliary tract cancers (C23–C24), head and neck cancers (C00–C14), brain cancer (C71), liver cancer (C22), pancreatic cancer (C25), lung cancer (C34), bone and articular cartilage cancers (C40–C41), breast cancer (C50), female genital organ cancers (C51–C58), male genital organ cancers (C60–C63), urinary tract cancers (C64–C68), thyroid cancer (C73), and lymphoma and leukaemia (C81–C96).

### Collected covariates

The primary demographic variables of the patients were age and gender. Socioeconomic status was determined on the basis of household income, residence and employment status (including self-employment). Residences were classified as urban (Seoul and other large cities) or rural (all other regions). Household income was divided into five groups, one of which comprised individuals engaged in a medical aid programme, whereas the remaining groups followed a four-quartile distribution. Individuals unable to afford insurance are classified by the government as eligible for medical assistance.

To evaluate the occurrence of comorbidities, the Charlson Comorbidity Index was constructed using the most frequently occuring ICD-10 codes from the NHIS database (Supplementary Table 2). In South Korea, people must declare any disability in the NHIS database in order to be eligible for social welfare benefits. Thus, information about underlying disabilities was also gathered. In South Korea, specialists in relevant professions conduct disability evaluations. A qualified physician assesses the impact of a condition on everyday life, and it is classified as mild to moderate or severe. Supplementary Table 3 provides the full classification of disabilities.

### Statistical methodology

Continuous variables were assessed through calculation of means and standard deviations. Categorical variables are expressed in terms of percentages and absolute values. To reduce the variation in variables between the psychiatric disorder and non-psychiatric-disorder groups, we performed propensity score matching (a method to decrease bias in observational research^14^) via the nearest neighbour method, with a calliper width of 0.25 and a 1:1 ratio without replacement. In the propensity score modelling, age, gender, residence, household income level, underlying disability, Charlson Comorbidity Index and underlying diseases were included. Following propensity score matching, the absolute standardised mean difference (ASD) was used to determine the balance between the two groups. An ASD of less than 0.1 was considered to indicate proper propensity score matching.

Cox regression analysis was performed on the propensity-score-matched cohort to compare the frequency of newly diagnosed cancers between the psychiatric disorder and non-psychiatric-disorder groups. An event was defined as a cancer diagnosis made on or after 1 January 2018. Time was defined as the period between 1 January 2018 and the diagnosis. In addition, a time-lag analysis was performed in the propensity-score-matched cohort to determine whether psychiatric disorders preceded the cancer or were diagnosed in response to early symptoms. Herein, a cancer diagnosis made on or after 1 January 2019 was considered as an event. A hazard plot for cancer diagnoses in the propensity-score-matched cohort was constructed using Kaplan–Meier estimation, and the log-rank test was used to compare the cumulative incidence of total cancer diagnoses between the two groups.

A sensitivity analysis was performed using multivariable Cox regression modelling to compare the results for the propensity-score-matched group with those for the entire cohort. The model incorporated all covariates and was adjusted for several factors. To enhance our understanding of the risks associated with a cancer diagnosis, we conducted further multivariable Cox regression analyses to compare individuals diagnosed with two, three or more psychiatric conditions with those diagnosed with only one psychiatric disorder. Additional multivariable Cox regression analyses were performed to investigate whether the likelihood of a cancer diagnosis differed among groups with specific psychiatric disorders.

Subgroup analyses were conducted on the basis of gender and age. Log–log plots were used to verify adherence to the foundational assumptions of the Cox proportional hazards models. The results are presented as hazard ratios accompanied by 95% confidence intervals. The multivariable model demonstrated no multicollinearity among the variables, as indicated by a variance inflation factor of 2. For the statistical analyses, we used R version 4.0.3 for Windows (R Foundation for Statistical Computing, Vienna, Austria; see https://cran.r-project.org/bin/windows/base/old/4.0.3/), with the significance threshold set to *P* < 0.05. The ‘MatchIt’ and ‘survival’ R packages were used for propensity score matching and survival analyses, respectively. The Coxph and Surv(time, event) functions in the ‘survival’ package were used for the Cox hazard modelling.

## Results

### Study population

**Fig. 1:**
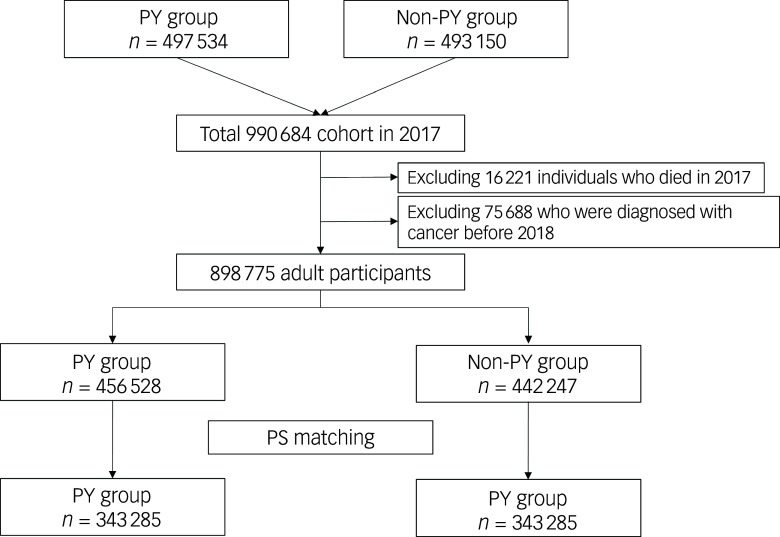
Flowchart illustrating the patient selection process for this study. PY, psychiatric disorder; PS, propensity score.

Figure 1 presents a flowchart representing the patient selection process. In 2017, 497 534 adults in the psychiatric disorder group were screened and compared with 493 150 individuals in the non-psychiatric-disorder group. After excluding 16 221 individuals who died in 2017 and 75 688 who were diagnosed with cancer before 2018, the study included 898 775 adult participants. After 1:1 propensity score matching, the final analysis included data of 686 570 adults (343 285 in each group). Table 1 displays the clinicopathological characteristics of patients in the psychiatric disorder and non-psychiatric-disorder groups before and after propensity score matching. All ASDs in the propensity-score-matched cohort were below 0.1, indicating a well balanced comparison between the two groups. Supplementary Fig. 1 illustrates the distributions of propensity scores before and after matching, showing that the distributions were comparable post-implementation.

**Table 1:** Clinicopathological characteristics of patients in the psychiatric disorder and non-psychiatric-disorder groups before and after propensity score matching

Variable	Entire cohort (*n* = 898 775)		ASD	Propensity-score-matched cohort (*n* = 686 570)		ASD
	Psychiatric disorder group *n* = 456 528	Non-psychiatric-disorder group *n* = 442 247		Psychiatric disorder group *n* = 343 285	Non-psychiatric-disorder group *n* = 343 285	
Age, years	58.2 (17.7)	58.4 (17.6)	0.008	57.7 (17.5)	56.6 (18.3)	0.057
Gender: male	164 991 (37.3)	174 035 (38.1)	0.002	128 547 (37.4)	129 067 (37.6)	0.006
Having a job	268 587 (60.7)	302 752 (66.3)	0.116	219 342 (63.9)	223 759 (65.2)	0.036
Residence						
Urban area	187 286 (42.3)	201 409 (44.1)		148 486 (43.3)	148 997 (43.4)	
Rural area	254 961 (57.7)	255 119 (55.9)	0.037	194 799 (56.7)	194 288 (56.6)	0.007
Household income level						
Medical aid programme group	41 249 (9.3)	85 817 (18.8)		18 055 (5.3)	14 647 (4.3)	
Q1	80 843 (18.3)	85 712 (18.8)	0.032	64 534 (18.8)	64 994 (18.9)	0.008
Q2	77 155 (17.4)	106 590 (23.3)	0.043	63 552 (18.5)	64 371 (18.8)	0.013
Q3	96 222 (21.8)	156 091 (34.2)	0.048	78 201 (22.8)	79 578 (23.2)	0.007
Q4	140 504 (31.8)	15 156 (3.3)	0.203	113 707 (33.1)	114 387 (33.3)	0.047
Unknown	6274 (1.4)	7162 (1.6)	0.013	5236 (1.5)	5308 (1.5)	0.003
Underlying disability						
Mild to moderate	33 661 (7.6)	24 792 (5.4)	0.086			0.027
Severe	29 551 (6.7)	10 317 (2.3)	0.174			0.040
CCI	2.1 (2.0)	1.2 (1.5)	0.450	1.6 (1.7)	1.4 (1.6)	0.099
Myocardial infarction	8891 (2.0)	4513 (1.0)	0.080	5138 (1.5)	4040 (1.2)	0.022
Congestive heart failure	35 099 (7.9)	17 767 (3.9)	0.157	20 355 (5.9)	16 143 (4.7)	0.044
Peripheral vascular disease	84 018 (19.0)	46 305 (10.1)	0.229	51 081 (14.9)	43 166 (12.6)	0.057
Cerebrovascular disease	74 059 (16.7)	30 115 (6.6)	0.278	38 734 (11.3)	29 459 (8.6)	0.071
Dementia	51 545 (11.7)	19 591 (4.3)	0.230	26 279 (7.7)	19 212 (5.6)	0.060
Chronic pulmonary disease	149 937 (33.9)	101 039 (22.1)	0.252	99 305 (28.9)	91 587 (26.7)	0.048
Rheumatic disease	26 486 (6.0)	13 910 (3.0)	0.127	16 026 (4.7)	13 082 (3.8)	0.031
Peptic ulcer disease	131 912 (29.8)	73 382 (16.1)	0.305	80 117 (23.3)	71 134 (20.4)	0.057
Mild liver disease	133 265 (30.1)	74 784 (16.4)	0.305	81 149 (23.6)	71 718 (20.9)	0.061
Diabetes mellitus without chronic complications	113 667 (25.7)	75 600 (16.6)	0.221	73 154 (21.3)	65 461 (19.1)	0.052
Diabetes mellitus with chronic complications	38 467 (8.7)	23 843 (5.2)	0.131	24 207 (7.1)	20 724 (6.0)	0.036
Hemiplegia or paraplegia	8128 (1.8)	2937 (0. 6)	0.089	4062 (1.2)	2797 (0.8)	0.023
Renal disease	10 895 (2.5)	6404 (1.4)	0.071	6671 (1.9)	5492 (1.6)	0.020
Moderate or severe liver disease	1817 (0.4)	610 (0.1)	0.047	871 (0.3)	602 (0.2)	0.011
AIDS/HIV	286 (0.1)	126 (0.1)	0.015	161 (0.0)	123 (0.0)	0.007

ASD, absolute standardised mean difference; CCI, Charlson comorbidity index; HIV, human immunodeficiency virus.

### Analyses used in the propensity-score-matched cohort

**Fig. 2:**
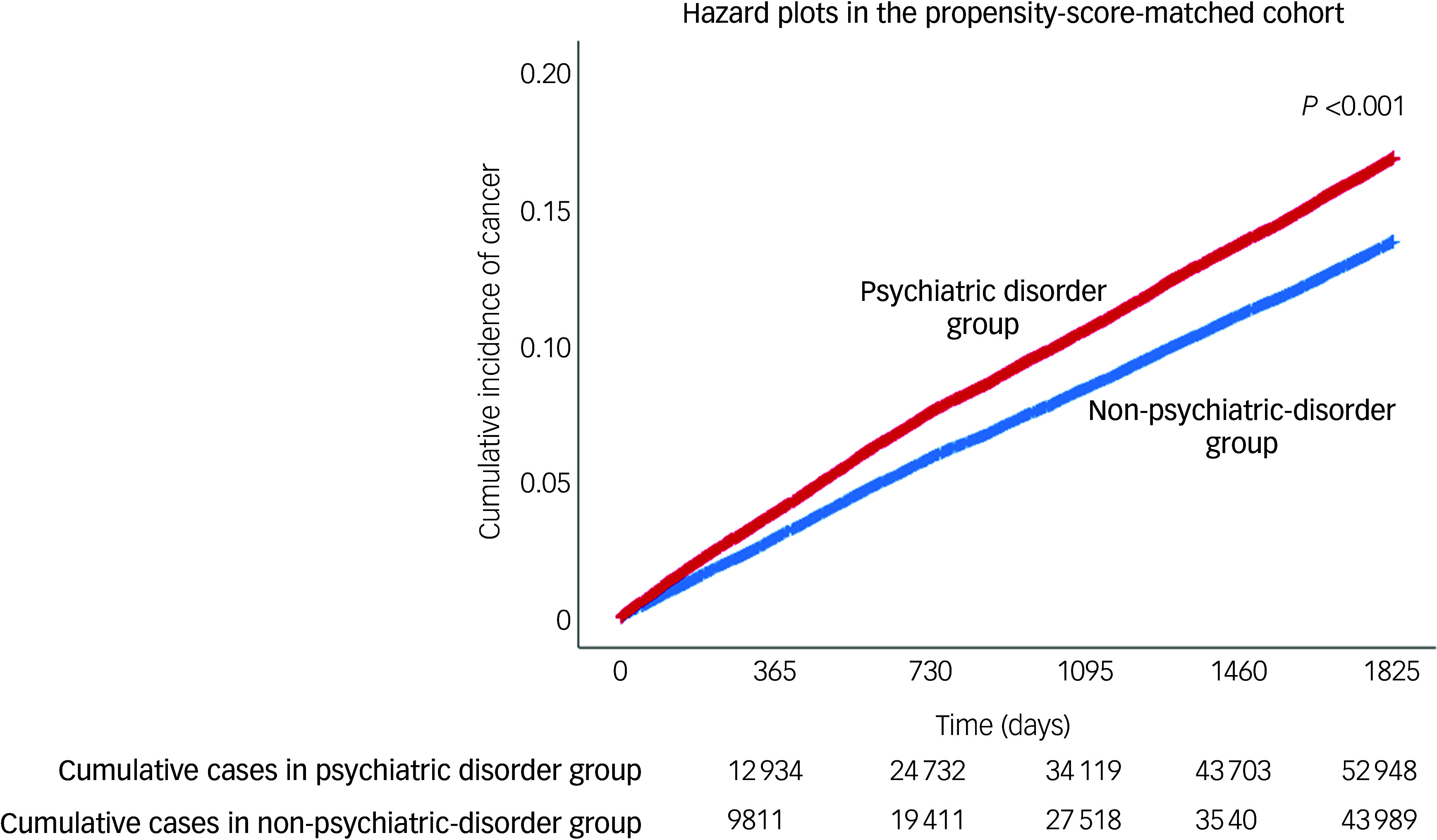
Hazard plots for cancer diagnoses in propensity-score-matched cohort.

Table 2 displays the results of the analysis for the propensity-score-matched cohort. After propensity score matching, the incidence of total cancer in the psychiatric disorder group during 2018–2022 was 15.4% (52 948/343 285), whereas that in the non-psychiatric-disorder group was 12.8% (43 989/343 285). In the Cox regression analysis, the psychiatric disorder group had a 23% (hazard ratio: 1.23, 95% CI: 1.21, 1.24; *P* < 0.001) higher incidence of total cancer than the non-psychiatric-disorder group. The psychiatric disorder group also exhibited increased incidence rates of brain cancer, breast cancer, colorectal cancer, female genital organ cancer, gastric cancer, gallbladder and biliary tract cancer, head and neck cancer, liver cancer, lung cancer, lymphoma or leukaemia, male genital organ cancer, pancreatic cancer, thyroid cancer and urinary tract cancer. Supplementary Table 4 presents the results of the time-lag analysis in the propensity-score-matched cohort. In the Cox regression analysis, the incidence of total cancer was 20% higher in the psychiatric disorder group (hazard ratio: 1.20, 95% CI: 1.18, 1.21; *P* < 0.001) than in the non-psychiatric-disorder group. Figure 2 shows hazard plots for cancer diagnoses in the propensity-score-matched cohort, with the psychiatric disorder group showing a higher cumulative incidence of total cancer than the non-psychiatric-disorder group (*P* < 0.001).

**Table 2:** Analysis results for the propensity-score-matched cohort

Outcome	*N* (event, %)	Hazard ratio (95% CI)	*P*-value
Total cancer			
Non-psychiatric-disorder group	43 989/343 285 (12.8)	1	
Psychiatric disorder group	52 948/343 285 (15.4)	1.23 (1.21, 1.24)	<0.001
Bone and cartilage cancer			
Non-psychiatric-disorder group	135/343 285 (0.0)	1	
Psychiatric disorder group	164/343 285 (0.0)	1.24 (0.98, 1.55)	0.069
Brain cancer			
Non-psychiatric-disorder group	384/343 285 (0.1)	1	
Psychiatric disorder group	495/343 285 (0.1)	1.31 (1.15, 1.50)	<0.001
Breast cancer			
Non-psychiatric-disorder group	2659/343 285 (0.8)	1	
Psychiatric disorder group	3001/343 285 (0.9)	1.15 (1.09, 1.21)	<0.001
Colorectal cancer			
Non-psychiatric-disorder group	8304/343 285 (2.4)	1	
Psychiatric disorder group	10 383/343 285 (3.0)	1.27 (1.24, 1.31)	<0.001
Oesophageal cancer			
Non-psychiatric-disorder group	239/343 285 (0.1)	1	
Psychiatric disorder group	267/343 285 (0.1)	1.14 (0.95, 1.35)	0.152
Female genital organ cancer			
Non-psychiatric-disorder group	5640/343 285 (1.6)	1	
Psychiatric disorder group	6760/343 285 (2.0)	1.22 (1.18, 1.26)	<0.001
Gastric cancer			
Non-psychiatric-disorder group	2977/343 285 (0.9)	1	
Psychiatric disorder group	3317/343 285 (1.0)	1.13 (1.08, 1.19)	<0.001
Gall bladder or bile duct cancer			
Non-psychiatric-disorder group	1686/343 285 (0.5)	1	
Psychiatric disorder group	1967/343 285 (0.6)	1.19 (1.11, 1.27)	<0.001
Head and neck cancer			
Non-psychiatric-disorder group	443/343 285 (0.1)	1	
Psychiatric disorder group	502/343 285 (0.1)	1.15 (1.01,1.31)	0.029
Liver cancer			
Non-psychiatric-disorder group	8608/343 285 (2.5)	1	
Psychiatric disorder group	10 703/343 285 (3.1)	1.27 (1.23, 1.30)	<0.001
Lung cancer			
Non-psychiatric-disorder group	5817/343 285 (1.7)	1	
Psychiatric disorder group	7073/343 285 (2.1)	1.24 (1.19, 1.28)	<0.001
Lymphoma or leukaemia			
Non-psychiatric-disorder group	2433/343 285 (0.7)	1	
Psychiatric disorder group	2741/343 285 (0.8)	1.15 (1.06, 1.21)	<0.001
Male genital organ cancer			
Non-psychiatric-disorder group	7423/343 285 (2.2)	1	
Psychiatric disorder group	9811/343 285 (2.9)	1.34 (1.30, 1.39)	<0.001
Pancreatic cancer			
Non-psychiatric-disorder group	8271/343 285 (2.4)	1	
Psychiatric disorder group	10 491/343 285 (3.1)	1.29 (1.26, 1.33)	<0.001
Thyroid cancer			
Non-psychiatric-disorder group	4127/343 285 (1.2)	1	
Psychiatric disorder group	4697/343 285 (1.4)	1.16 (1.11, 1.21)	<0.001
Urinary tract cancer			
Non-psychiatric-disorder group	3680/343 285 (1.1)	1	
Psychiatric disorder group	4679/343 285 (1.4)	1.29 (1.24, 1.35)	<0.001

### Analyses in the entire cohort

Table 3 displays the outcomes of the multivariable Cox regression model for all cancer diagnoses within the entire cohort. With model 1, the psychiatric disorder group had a 19% (hazard ratio: 1.19, 95% CI: 1.18, 1.21; *P* < 0.001) higher incidence of total cancer than the non-psychiatric-disorder group. With model 3, compared with the non-psychiatric-disorder group, individuals with two or more psychiatric disorders and those with three or more psychiatric disorders had 27% (hazard ratio: 1.27, 95% CI: 1.24, 1.29; *P* < 0.001) and 31% (hazard ratio: 1.31, 95% CI: 1.27, 1.35; *P* < 0.001) higher incidence rates of total cancer, respectively.

**Table 3:** Multivariable Cox regression model for total cancer incidence

Variable	Hazard ratio (95% CI)	*P*-value
Model 1		
Non-psychiatric-disorder group (*n* = 442 247)	1	
Psychiatric disorder group (*n* = 456 528)	1.19 (1.18, 1.21)	<0.001
Model 2		
Non-psychiatric-disorder group (*n* = 456 528)	1	
One psychiatric disorder group (*n* = 310 107)	1.17 (1.15, 1.18)	<0.001
Two or more psychiatric disorders group (*n* = 132 140)	1.27 (1.25, 1.30)	<0.001
Model 3		
Non-psychiatric-disorder group (*n* = 456 528)	1	
One psychiatric disorder group (*n* = 310 107)	1.17 (1.15, 1.18)	<0.001
Two psychiatric disorders group (*n* = 101 528)	1.27 (1.24, 1.29)	<0.001
Three or more psychiatric disorders group (*n* = 30 612)	1.31 (1.27, 1.35)	<0.001
Model 4		
ADHD (*n* = 2923)	0.68 (0.58, 0.79)	<0.001
Anorexia nervosa (*n* = 2636)	0.97 (0.88, 1.08)	0.608
Alcohol-related disorder (*n* = 16 628)	1.27 (1.23, 1.32)	<0.001
Anxiety disorder (*n* = 306 295)	1.15 (1.14, 1.17)	<0.001
Autism (*n* = 968)	0.57 (0.41, 0.78)	<0.001
Bipolar disorder (*n* = 41 388)	0.94 (0.91, 1.05)	0.402
Major depressive disorder (*n* = 206 669)	1.16 (1.15, 1.18)	<0.001
OCD (*n* = 5290)	0.97 (0.90, 1.05)	0.468
Schizophrenia (*n* = 28 880)	0.93 (0.90, 0.97)	<0.001
Tic (*n* = 1186)	0.66 (0.53, 0.84)	<0.001

ADHD, attention-deficit hyperactivity disorder; OCD, obsessive–compulsive disorder.

Model 4 showed significant associations between an increased incidence of total cancer and specific psychiatric disorders among individuals with alcohol-related diseases (hazard ratio: 1.27, 95% CI: 1.23, 1.32; *P* < 0.001), anxiety disorder (hazard ratio: 1.15, 95% CI: 1.14, 1.17; *P* < 0.001) and major depressive disorder (hazard ratio: 1.16, 95% CI: 1.15, 1.18; *P* < 0.001). Supplementary Table 5 presents all hazard ratios with 95% CIs from multivariable model 1.

### Subgroup analyses

Table 4 presents the findings from the subgroup analyses. The psychiatric disorder group exhibited a higher risk of total cancer incidence than the non-psychiatric-disorder group in both the male (hazard ratio: 1.22, 95% CI: 1.19, 1.24; *P* < 0.001) and female (hazard ratio: 1.18, 95% CI: 1.17, 1.20; *P* < 0.001) subgroups. Furthermore, the psychiatric disorder group showed an increased risk of death by suicide compared with the non-psychiatric-disorder group across age groups (18–40 years, hazard ratio: 1.25, 95% CI: 1.19, 1.30; *P* < 0.001; 41–60 years, hazard ratio: 1.24, 95% CI: 1.21, 1.27; *P* < 0.001; and ≥61 years, hazard ratio: 1.15, 95% CI: 1.13, 1.17; *P* < 0.001).

**Table 4:** Subgroup analyses for total cancer incidence

Variable	Hazard ratio (95% CI)	*P*-value
Gender: male		
Non-psychiatric-disorder group	1	
Psychiatric disorder group	1.22 (1.19, 1.24)	<0.001
Gender: female		
Non-psychiatric-disorder group	1	
Psychiatric disorder group	1.18 (1.17, 1.20)	<0.001
Age: 18–40 years		
Non-psychiatric-disorder group	1	
Psychiatric disorder group	1.25 (1.19, 1.30)	<0.001
Age: 41–60 years		
Non-psychiatric-disorder group	1	
Psychiatric disorder group	1.24 (1.21, 1.27)	<0.001
Age: ≥61 years		
Non-psychiatric-disorder group	1	
Psychiatric disorder group	1.15 (1.13, 1.17)	<0.001

## Discussion

The results of this population-based cohort study indicate that individuals with psychiatric disorders have an increased risk of developing cancer compared with those without such disorders. This risk appears to increase with the presence of multiple diagnoses of psychiatric disorders. Among mental health conditions, alcohol-related disorders, anxiety disorders and major depressive disorder show particular associations with cancer, highlighting the need for policies to facilitate early detection and screening in individuals with these conditions.

Anxiety disorders, which were the most common psychiatric condition among participants in this study, are linked to an increased incidence of cancer. Psychological factors may affect immune and endocrine functions, hence the longstanding hypothesis that psychosocial elements play a part in the occurrence of cancer.^15^ Accumulating evidence suggests that the immune system connects the central nervous system and disease mechanisms,^16^ and anxiety disorders are characterised by impaired cellular immunological activity, which may be associated with cancer-related mechanisms.^17^ Indeed, several studies have shown that external factors such as stress, depression and social support significantly affect the immune system, with a subsequent influence on the onset and progression of cancer.^18,19^

Similarly, individuals with major depressive disorder have been reported to have a higher incidence of cancer; for instance, a small positive association has been reported between depression and the overall risk of developing cancer, including specific risks for liver and lung cancers.^20^ The origins of cancer are multifaceted and involve a combination of genetic predispositions, lifestyle choices, environmental influences, and psychosocial factors such as depression. Since the 1980s, research has indicated that depression has effects on immune and endocrine functions, cancer metastasis, treatment tolerance and various other processes.^21,22^ Moreover, prospective epidemiological studies suggest that depression may elevate the risk of developing cancer,^23^ and a recent retrospective cohort study found that depression was associated with an elevated cancer risk, varying from 10 to 39%, depending on the cancer type.^24^

Depression and cancer are both linked to increased inflammation, which activates the hypothalamic–pituitary–adrenal axis, influencing multiple physiological functions including metabolic processes and stress response mechanisms. Individuals diagnosed with depression have a higher prevalence of chronic illnesses linked to pain and inflammation compared with those without such a diagnosis.^25^ Furthermore, persistent inflammation has been shown to increase the risk of cancer.^26^ Downstream effectors of the hypothalamic–pituitary–adrenal pathway include pro-inflammatory elements such as cytokines; these may be upregulated or downregulated, with consequent effects on anti-cancer immune cells that promote the spread of cancer.^27^

Importantly, alcohol-related disorders are associated with a higher incidence of cancer. Numerous mechanistic pathways have been identified through which alcohol intake, specifically ethanol intake, contributes to cancer development, although certain aspects remain incompletely elucidated. Ethanol and acetaldehyde metabolites have the potential to induce DNA damage and hinder DNA synthesis and repair.^28^ In addition, both ethanol and acetaldehyde interfere with DNA methylation.^29^ Ethanol can trigger inflammation and oxidative stress, resulting in lipid peroxidation and additional DNA damage.^28^ The International Agency for Research on Cancer classifies alcohol as a group 1 carcinogen that is causally associated with seven cancer types: oesophageal, liver, colorectal and breast cancer,^30^ and 740 000 new annual cancer cases worldwide are linked to alcohol consumption.^31^ Recently, there has been some debate regarding the differences in cancer risk between light and heavy drinkers, with the evidence remaining inconclusive.^32^

This study had a few limitations. First, it did not include information on lifestyle factors such as alcohol and tobacco use, which could significantly increase the risk of cancer. Second, we did not obtain information on hormonal changes or genetic mutations due to the use of psychiatric drugs. Third, although this was a large cohort study, the data were gathered retrospectively, potentially introducing unmeasured confounders and biases. Furthermore, the findings of this study were derived from data collected in South Korea and may not be applicable to other populations. Fourth, we did not perform an analysis of cancer staging at diagnosis, which may have indicated whether patients with psychiatric diseases received later diagnoses; such healthcare disparities are an important issue for patients with cancer.^33^ Fifth, family history of cancer was not included as a covariate in this study. Last, we did not consider psychiatric medication use, which may influence metabolic and immune function. However, despite these limitations, we found that patients with psychiatric disorders had a higher risk of cancer than those without psychiatric disorders, and this risk seems to be increasing with the rising incidence of psychiatric disorders. Specifically, the results of this study demonstrated associations of alcohol-related disorders, anxiety disorders and major depressive disorder with cancer. Future studies should thus take lifestyle factors into account.

## Supporting information

Oh et al. supplementary material 1 Oh et al. supplementary material

Oh et al. supplementary material 2 Oh et al. supplementary material

Oh et al. supplementary material 3 Oh et al. supplementary material

Oh et al. supplementary material 4 Oh et al. supplementary material

Oh et al. supplementary material 5 Oh et al. supplementary material

Oh et al. supplementary material 6 Oh et al. supplementary material

## Data Availability

The data that support the findings of this study are available on request from the corresponding author, I.-A.S.

## Supplementary material

The supplementary material is available online at https://doi.org/10.1192/bjo.2025.783
