# Supplementary figures and images for: Cancer risk in individuals with psychiatric disorders: population-based cohort study
Source: BJPsych Open. 2025 Jun 20;11(4):e122. doi: 10.1192/bjo.2025.783 (PMC12188226; doi:10.1192/bjo.2025.783)

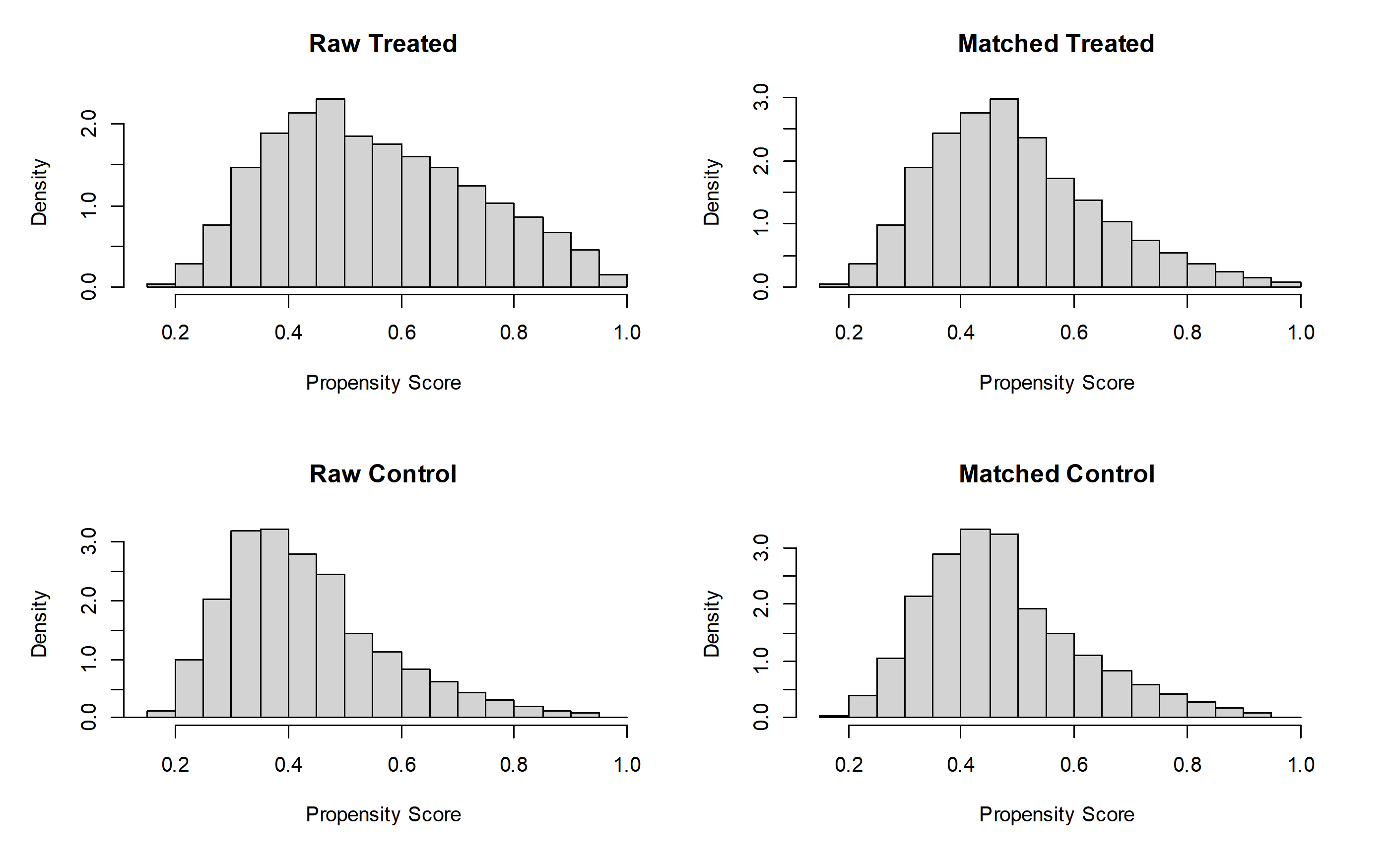

Supplement: Oh et al. supplementary material 1 — Oh et al. supplementary material [file S2056472425007835sup001.tif]
