# Supplementary material for: Cancer risk in individuals with psychiatric disorders: population-based cohort study
Source: BJPsych Open. 2025 Jun 20;11(4):e122. doi: 10.1192/bjo.2025.783 (PMC12188226; doi:10.1192/bjo.2025.783)
Supplement: Oh et al. supplementary material 2 — Oh et al. supplementary material [file S2056472425007835sup002.docx]

Table S1. ICD-10 codes of psychiatric disorders

**Schizophrenia (SCZ) F20, F23; F23.2; F25,**

**Bipolar Disorder (BIP) (F30 and F31)**

**Autism (ASD) (F84.0, F84.1, F84.5 & F84.9),**

**Anorexia Nervosa (ANO) (F50.0**)

**Alcohol related disorder (F10)**

**Attention Deficit Hyperactivity Disorder (ADHD) (F90**)

**OCD (F42**)

**Major Depressive Disorder (MDD) (F32 & F33)**

**Tic Disorder (TIC):** F95

**Anxiety disorder:** F40, F41
