# Supplementary material for: Cancer risk in individuals with psychiatric disorders: population-based cohort study
Source: BJPsych Open. 2025 Jun 20;11(4):e122. doi: 10.1192/bjo.2025.783 (PMC12188226; doi:10.1192/bjo.2025.783)
Supplement: Oh et al. supplementary material 5 — Oh et al. supplementary material [file S2056472425007835sup005.docx]

Table S4. Time-lag analysis in the PS-matched cohort.

| Outcome | | | N (event, %) | HR (95% CI) | *P*-value |
| --- | --- | --- | --- | --- | --- |
| Total cancer | | |  |  |  |
|  | Non-PY group | | 35,820/343,285 (10.4) | 1 |  |
|  | | PY group | 41,972/343,285 (12.2) | 1.20 (1.18, 1.21) | <0.001 |

PS, propensity score; HR, hazard ratio; CI, confidence interval; PY, psychiatric disorder
