# Supplementary material for: Cancer risk in individuals with psychiatric disorders: population-based cohort study
Source: BJPsych Open. 2025 Jun 20;11(4):e122. doi: 10.1192/bjo.2025.783 (PMC12188226; doi:10.1192/bjo.2025.783)
Supplement: Oh et al. supplementary material 6 — Oh et al. supplementary material [file S2056472425007835sup006.docx]

Table S5. All HRs with 95% CIs in multivariable Model 1

| Variable | | HR (95% CI) | *P*-value |
| --- | --- | --- | --- |
| Age, year | | 1.02 (1.02, 1.02) | <0.001 |
| Male sex | | 1.35 (1.34, 1.37) | <0.001 |
| Having a job | | 0.99 (0.98, 1.00) | 0.042 |
| Residence | |  |  |
|  | Urban area | 1 |  |
|  | Rural area | 0.97 (0.96, 0.98) | <0.001 |
| Household income level | |  |  |
|  | Medical aid program group | 1.10 (1.07, 1.13) | <0.001 |
|  | Q1 | 1 |  |
|  | Q2 | 1.00 (0.98, 1.02) | 0.773 |
|  | Q3 | 1.00 (0.98, 1.02) | 0.785 |
|  | Q4 | 1.02 (1.00, 1.04) | 0.013 |
|  | Unknown | 1.06 (1.01, 1.11) | 0.013 |
| Underlying disability | |  |  |
|  | Mild to moderate | 1.07 (1.045, 1.09) | <0.001 |
|  | Severe | 0.97 (1.00, 1.02) | 0.067 |
| Charlson comorbidity index, 1 point | | 1.17 (1.14, 1.20) | <0.001 |
|  | Myocardial infarction | 1.06 (1.02, 1.10) | 0.005 |
|  | Congestive heart failure | 1.01 (0.99, 1.03) | 0.542 |
|  | Peripheral vascular disease | 1.04 (1.03, 1.06) | <0.001 |
|  | Cerebrovascular disease | 1.06 (1.04, 1.08) | <0.001 |
|  | Dementia | 0.75 (0.73, 0.76) | <0.001 |
|  | Chronic pulmonary disease | 1.17 (1.16, 1.19) | <0.001 |
|  | Rheumatic disease | 1.14 (1.11, 1.17) | <0.001 |
|  | Peptic ulcer disease | 1.17 (1.15, 1.18) | <0.001 |
|  | Mild liver disease | 1.27 (1.26, 1.29) | <0.001 |
|  | Diabetes without chronic complication | 1.15 (1.14, 1.17) | <0.001 |
|  | Diabetes with chronic complication | 1.15 (1.13, 1.17) | <0.001 |
|  | Hemiplegia or paraplegia | 0.82 (0.78, 0.86) | <0.001 |
|  | Renal disease | 1.12 (1.08, 1.15) | <0.001 |
|  | Moderate or severe liver disease | 1.55 (1.44, 1.67) | <0.001 |
|  | AIDS/HIV | 1.27 (1.03, 1.57) | 0.023 |

HR, hazard ratio; CI, confidence interval; DM, diabetes mellitus; AIDS, Acquired immunodeficiency syndrome; HIV, human immunodeficiency virus
